# Impact of simulation-based training in surgical chest tube insertion on a model of traumatic pneumothorax

**DOI:** 10.1186/s41077-016-0021-2

**Published:** 2016-06-10

**Authors:** Alexandre Léger, Aiham Ghazali, Franck Petitpas, Youcef Guéchi, Amélie Boureau-Voultoury, Denis Oriot

**Affiliations:** 1 Pediatric Department, Basse-Terre Medical Center, Guadeloupe, France; 2 Emergency Department, Pitié-Salpétrière University Hospital, Paris, France; 3 Simulation Laboratory, Faculty of Medicine, University of Poitiers, Poitiers, France; 4 Surgical Intensive Care Unit, University Hospital, Poitiers, France; 5 Emergency Department, University Hospital, Poitiers, France; 6 Pediatric Emergency Department, University Hospital, 2 rue de la Milétrie, 86000 Poitiers, France

**Keywords:** Simulation-based education, Trauma, Pneumothorax, Chest tube, Assessment, Success rate, Performance, Task trainer

## Abstract

**Background:**

Chest tube insertion is required for most cases of traumatic pneumothorax. However, this procedure entails risks of potentially life-threatening complications. A “surgical” approach is widely recommended to minimize these risks. Simulation-based education has previously been used in surgical chest tube insertion, but not been subjected to rigorous evaluation.

**Methods:**

The primary objective was to evaluate the success rate of surgical chest tube insertion in a task trainer (previously published). Secondary objectives were to assess performance with a performance assessment scale (previously designed), to measure the time of insertion, and to seek out a correlation between the learner’s status, experience, and performance and success rate. Participants were surveyed for realism of the model and satisfaction; 65 participants (18 residents, 47 senior physicians) were randomized into SIM+ or SIM− groups. Both groups received didactic lessons. The SIM+ group was assigned deliberate practice on the model under supervision. Both groups were assessed on the model 1 month later.

**Results:**

There was no difference between the SIM+ (*n* = 34) and SIM− (*n* = 31) groups regarding status (*p* = 0.44) or previous surgical insertion (*p* = 0.12). Success rate was 97 % (SIM+) and 58 % (SIM−), *p* = 0.0002. Performance score was 16.29 ± 1.82 (SIM+) and 11.39 ± 3.67 (SIM−), *p* = 3.13 × 10^−8^. SIM+ presented shorter dissection time than SIM− (*p* = 0.047), but procedure time was similar (*p* = 0.71). Status or experience was not correlated with success rate, performance score, procedure time, or dissection time. SIM+ gained more self-confidence, judged the model more realistic, and were more satisfied than SIM−.

**Conclusions:**

Simulation-based education significantly improved the success rate and performance of surgical chest tube insertion on a traumatic pneumothorax model.

## Background

Insertion of a chest tube is a mandatory procedure for treatment of a significant traumatic pneumothorax [1, 2]. However, this procedure remains somewhat difficult, stressful, and at risk of potentially life-threatening complications [3–10]. Numerous physicians acknowledge their lack of training and feel uncomfortable carrying out a chest tube insertion, particularly in a young patient [11, 12]. Furthermore, many physicians may have difficulty supervising novices while inserting a chest tube with a surgical approach [13]. For three decades, simulation-based education has been developed, allowing learners to gain competence and develop their skills through performance in a safe environment, prior to practice in a clinical setting [14–20]. Nevertheless, no study to date has reported a rigorous evaluation of the benefit of simulation on surgical approach for chest tube insertion.

The aim of this study was to evaluate the benefit of simulation-based education on the performance and success rate of surgical insertion of a chest tube on a model of traumatic pneumothorax.

## Methods

### Study

This was a prospective, randomized, controlled, bicentric study, carried out from May 2013 to October 2013 in the Simulation Laboratory of the Faculty of Medicine of Poitiers and in the Medical Center of Cayenne, French Guyana, after approval by the Institutional Research Board (Scientific Committee of the Faculty of Medicine of Poitiers, file number 11–37). All participants signed an informed consent form. Results were kept anonymous.

### Objectives

The primary objective was to evaluate the success rate of surgical insertion of a chest tube in a task trainer simulator of traumatic pneumothorax.

The secondary objectives were (1) to assess the performance of insertion procedure according to a performance assessment scale, (2) to measure the global procedure time and dissection time during insertion, (3) to seek out a correlation between the learner’s status, experience, and performance and success rate, and (4) to survey the learners for their evaluation of the realism of the model, self-confidence, and satisfaction after simulation experience.

### Population

Sixty-five healthcare providers (18 residents and 47 senior emergency physicians), representing two groups of registered participants (Poitiers and Cayenne) for the pediatric emergency procedure course at the University of Poitiers (carried out in Poitiers and in Cayenne), were given a chance to participate.

### Intervention

Since the surgical approach is rarely taught and practiced in France, all participants received a 1-h academic lesson, prior to the simulation session, on current international recommendations for surgical chest tube insertion for traumatic pneumothorax [1, 21–24]. This didactic lesson was performed by three Advanced Trauma Life Support (ATLS)-certified supervisors. The session consisted in a presentation of the currently recommended safest approach to chest tube insertion in case of traumatic pneumothorax: landmarks on the medio-axillary line, in the fourth or fifth intercostal space [21, 22, 25], use of a tube without chuck or handle, dissection of chest wall muscular layers with a Kelly clamp, followed by insertion of a gloved finger probing into the chest cavity to confirm pleural placement, and strip any adhesions facing the insertion site [21]. In small children, it is recommended to tunnel the chest tube by a skin incision at the underlying intercostal space. This provides a subcutaneous path for securing the tube and avoids its falling out due to a thin chest wall [22].

### Comparison

Randomization was based on a list of 65 random numbers—even numbers for SIM+ and odd ones for SIM− group allocation. The SIM+ group participants (*n* = 34) were assigned for deliberate practice on the simulator just after the didactic lesson, whereas the SIM− group participants (*n* = 31) were not assigned any simulation practice. The SIM+ group participants had the opportunity to practice several times (mean = four times) on the simulator with immediate feedback from the supervisor. The SIM− group participants were exposed 5 min to the simulator and its environment (webcam, laptop, chest tube equipment) but did not perform chest tube insertions. One month later, all participants (SIM+ and SIM−) were evaluated during an assessment session on the simulator.

Every participant received a questionnaire on clinical experience in chest tube insertion—especially surgical approach—before the didactic lesson and during the month that followed prior to the assessment on the simulator. We arbitrarily distinguished experienced participants who had inserted at least five chest tubes (with a surgical approach) during the last 5 years from novices who had inserted less than five or none during the same span time.

At the end of the study, all participants were offered a chance for deliberate practice on the simulator with supervision.

### Outcomes

**Fig. 1:**
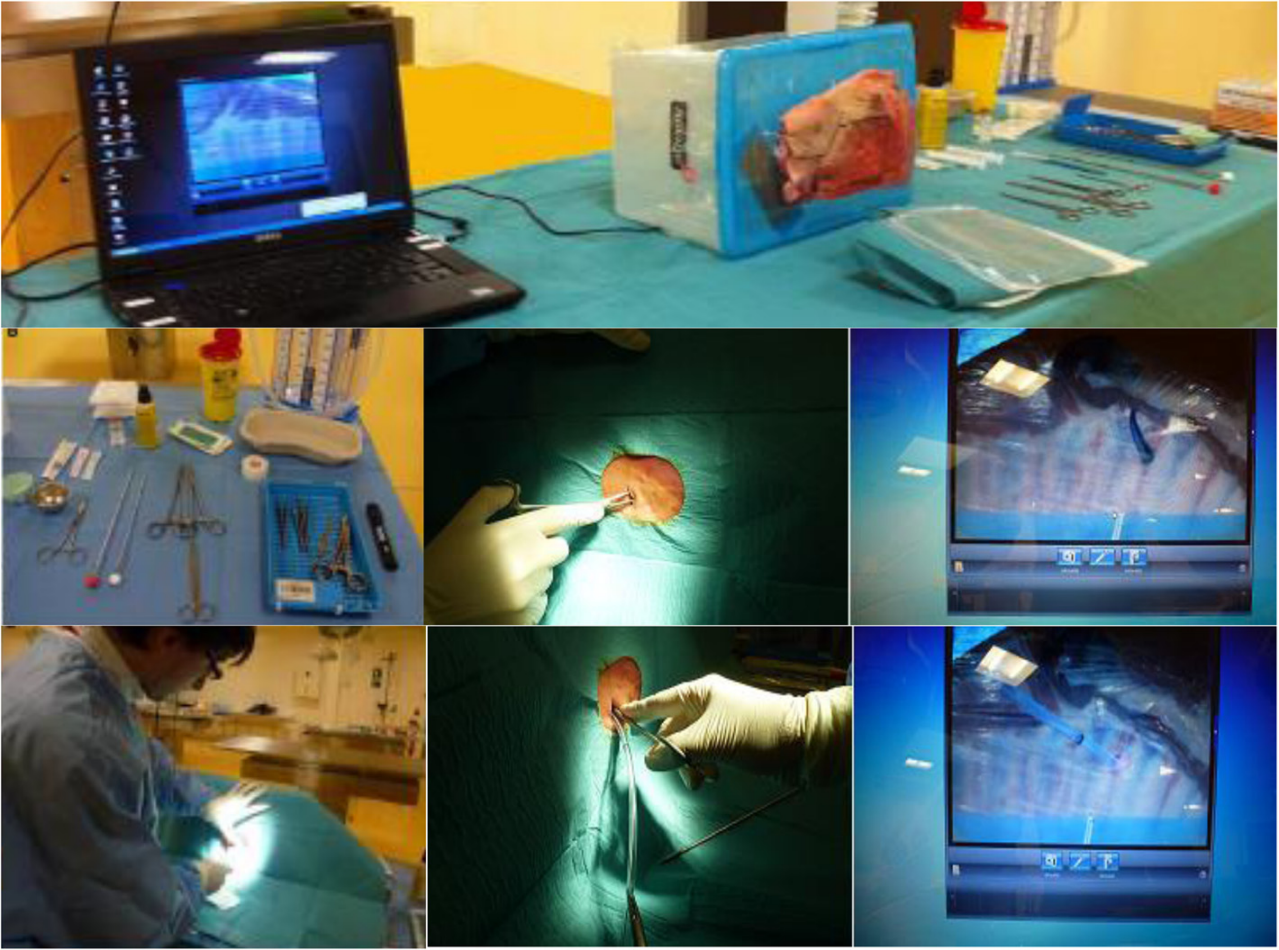
Assembled model (lamb half chest wall attached to the box cover) with a webcam in the back of the box connected to the laptop, set on the worktable for chest tube insertion procedure and video-controlled assessment; sterile equipment for chest tube insertion; extra- and intrathoracic views (webcam view on the laptop screen) of the introduction of the Kelly clamp with closed points through the parietal pleura; participant locating the landmarks for the incision site; extra- and intrathoracic views of the introduction of the chest tube with the Kelly clamp, directed upwards and forwards

The scenario consisted in an emergency surgical chest tube insertion for a traumatic pneumothorax in a teenager. The model was the one we had previously developed and published [26]. Briefly, the model assembled a lamb half chest tightly fixed on a box cover (Fig. 1). A spread-out two-layer plastic film simulated the pleural membranes. Inside the box, a webcam connected to a laptop made it possible to assess the intrathoracic steps of the procedure. All the required disposable equipment was set on a table: a 24-Fr-wide Joly drain (diameter 5.4 mm, length 40 cm, with two lateral holes, without chuck or handle) (Fig. 1). The landmark of the fourth rib was indicated to the participant. All simulation sessions were videotaped.

Success was assessed on the laptop connected to the webcam using two criteria: (1) sudden loss of resistance while passing through pleural membranes (one could hear the “pop” of the penetration in the pleural space) and (2) insertion of the chest tube without resistance over at least 10 cm (at or over the third black bar). Assessment of performance was carried out by an independent observer expert in surgical approach for chest tube insertion (ATLS-certified or ATLS instructor), who was neither a supervisor nor a research investigator. He was unaware of the breakdown into two groups. Four observers participated in the assessment (two emergency physicians, one intensivist, and one pediatric intensivist). All were trained for surgical chest tube insertion assessment according to a specific scale that we had previously designed and validated [27]. It included eight steps, detailing the procedure: asepsis, local anesthesia, cutaneous incision and dissection of the thoracic wall, confirmation of insertion, introduction of the chest tube with a Kelly clamp, securing the water seal tubing, securing the chest tube, and location of the incision site (which was examined by the observer after the procedure had been completed). Each item was ranked 1 (correctly performed) or 0 (not done or incorrectly performed). The maximum total score was over 20 points. This scale had acceptable internal coherence (Cronbach’s alpha coefficient = 0.747) and high inter-observer reproducibility (intra-class correlation coefficient = 0.966). Because of the webcam connected to a laptop inside the model, the observer could assess the performance of the extra- and intrathoracic steps of the procedure.

Timing of the different steps of the procedure was recorded. A stopwatch was started at the beginning of the procedure (*t*0) to record different times: beginning of the cutaneous incision (*t*1), chest tube passing through the skin (*t*2), connection of chest tube to water seal tubing (*t*3), and end of the procedure after securing the chest tube on the skin (*t*4). Dissection time was consequently defined as *t*2 − *t*1.

After each chest tube insertion, a debriefing was performed by the observer, and all the observers were trained in debriefing by good judgment [28]. Videotape replay during debriefing could render participants aware of their gaps in performance [29].

After each assessment, the participant was asked to fill out a questionnaire on realism of the model, self-confidence, and overall satisfaction with simulation-based education using 10-point Likert scales.

### Statistics

Analysis was carried out on Biostat TGV software and Excel 2010. Descriptive analysis included percentage, mean, and standard deviation (SD) of every variable. Comparative analysis used paired Student’s *t* test, with an ANOVA for repeated measures when necessary. Correlation between training and performance or success rate used Spearman’s test. Correlation between success and performance used Pearson’s test. A *p* value of <0.05 was considered as significant.

## Results

### Population studied

Among the 65 included participants, 47 were senior physicians (72.3 %) and 18 were residents (27.7 %). Twenty-three participants (only 49 % of senior physicians) were considered as experienced as they had previously surgically inserted more than five chest tubes during the previous 2 years. Thirty-four participants were randomized in the SIM+ group and 31 in the SIM− group, independently from their status and experience in chest tube insertion. There were no differences between the groups regarding status (*p* = 0.44) or previous experience in surgical chest tube insertion (*p* = 0.12) (Table 1). None of the participants had inserted chest tubes during the month having elapsed between the didactic lesson and the assessment.

**Table 1:** Comparison of SIM+ and SIM− groups according to status and previous clinical experience of participants

	SIM+ group (*n* = 34)	SIM− group (*n* = 31)	*p*
Residents	8 (23 %)	10 (32 %)	0.44
Senior physicians	26 (77 %)	21 (68 %)	
Novices (<5 drains)	25 (74 %)	17 (55 %)	0.12
Experienced (≥5 drains)	9 (26 %)	14 (45 %)	

### Primary objective

Mean success rate for surgical chest tube insertion was 78.5 % for the whole population. It was 97 % (33 drains/34) in the SIM+ group vs. 58 % (18 drains/31) in the SIM− group, *p* < 0.001 (Table 2).

**Table 2:** Comparison of success rate, performance score, procedure time, and dissection time according to group allocation, status, and previous clinical experience

Groups			
	SIM+ group (*n* = 34)	SIM− group (*n* = 31)	*p*
Success rate (%)	97 %	58 %	<0.001
Performance score (M ± SD)	16.29 ± 1.82	11.39 ± 3.67	<0.001
Procedure time (M ± SD)	10 min 19 s ± 2 min 19 s	10 min 7 s ± 2 min 9 s	0.71
Dissection time (M ± SD)	2 min 1 s ± 1 min 2 s	2 min 37 s ± 1 min 17 s	0.047
Status			
	Senior physicians (*n* = 47)	Residents (*n* = 18)	*p*
Success rate (%)	78.7 %	77.8 %	0.94
Performance score (M ± SD)	14.32 ± 3.58	13 ± 4.14	0.24
Procedure time (M ± SD)	10 min 20 s ± 2 min 17 s	9 min 57 s ± 2 min 5 s	0.52
Dissection time (M ± SD)	2 min 8 s ± 1 min 3 s	2 min 43 s ± 1 min 26 s	0.13
Previous experience			
	Experienced (*n* = 23)	Novices (*n* = 42)	*p*
Success rate (%)	82.6 %	76.2 %	0.54
Performance score (M ± SD)	14.22 ± 3.18	13.81 ± 4.07	0.66
Procedure time (M ± SD)	10 min 13 s ± 2 min 29 s	10 min 14 s ± 2 min 5 s	0.98
Dissection time (M ± SD)	2 min 13 s ± 1 min 7 s	2 min 21 s ± 1 min 14 s	0.65

*M* mean, *SD* standard deviation

### Secondary objectives

The mean performance assessment score was 13.95 ± 3.76 for the whole population. It was significantly higher in the SIM+ group (16.29 ± 1.82) than in the SIM− group (11.39 ± 3.67), *p* < 0.001 (Table 2). Details of scores per item of the scale showed that the typically surgical steps of the procedure were those with a significant difference between groups, i.e., location of the insertion site, incision and dissection, confirmation of location by probing a gloved finger into the pleural space, introduction of the chest tube with a Kelly clamp, and securing the tubing and chest tube (Table 3). Success was found to be correlated to performance with Pearson’s correlation coefficient of 0.85.

**Table 3:** Details of the scores (mean ± standard deviation) obtained at the different steps of the performance assessment scale for the SIM+ and SIM− groups

Steps of the scale (maximum score)	SIM+ *n* = 34	SIM− *n* = 31	*p*
Antiseptic procedure (3)	2.61 ± 0.65	2.36 ± 0.66	0.11
Location of incision site (1)	0.94 ± 0.23	0.69 ± 0.47	0.005
Local anesthesia (1)	0.88 ± 0.33	0.85 ± 0.37	0.61
Incision and dissection (6)	4.41 ± 0.70	2.43 ± 1.31	<0.001
Confirmation of location (2)	1.62 ± 0.69	0.98 ± 0.91	0.001
Introduction of chest tube with a Kelly clamp (4)	3.28 ± 1.03	2.46 ± 1.09	0.002
Securing water seal tubing (1)	0.81 ± 0.38	0.23 ± 0.42	<0.001
Securing chest tube (2)	1.74 ± 0.44	1.39 ± 0.61	0.01
Total (20)	16.29 ± 1.82	11.39 ± 3.67	<0.001

There was no difference in the procedure time between SIM+ and SIM−, *p* = 0.71. By contrast, the SIM+ group had a shorter dissection time than the SIM− group, *p* = 0.047. There were no correlations between status and procedure time (*p* = 0.52) or dissection time (*p* = 0.16), nor between a participant’s previous experience and procedure time (*p* = 0.98) or dissection time (*p* = 0.65) (Table 2). Status was not correlated with either performance score (*p* = 0.24) or success rate (*p* = 0.94).

There was no difference between experienced participants and novices regarding performance score (*p* = 0.66) or success rate (*p* = 0.54) (Table 2).

The SIM+ group participants gained more self-confidence than the SIM− ones (*p* = 0.036). The former judged the surgical chest tube insertion model with more realism (*p* = 0.0039) and were more satisfied with the simulation experience than the SIM− participants (8.21 ± 0.88 vs. 7.71 ± 0.74, *p* = 0.016) (Table 4).

**Table 4:** Comparison of the assessment of realism of the model, gain in self-confidence, and global satisfaction according to the group (on a 0–10 scale). Mean ± standard deviation

	SIM+ group (*n* = 34)	SIM− group (*n* = 31)	*p*
Realism of the model	7.65 ± 1.01	6.97 ± 0.83	0.0039
Gain in self-confidence	7.94 ± 0.69	7.55 ± 0.93	0.0364
Global satisfaction	8.21 ± 0.88	7.71 ± 0.74	0.0169

## Discussion

### Main results

On a traumatic pneumothorax model, simulation-based education was associated with higher success rate and performance score. These results were directly due to the simulation training, with no influence of status or previous clinical experience in surgical chest tube insertion. To our knowledge, this is the first study reporting the benefit of simulation-based education on success rate and performance for a surgical chest tube insertion.

### Limitations

This study nevertheless presented some limitations. All participants were registered for the pediatric emergency procedure university course, which could have represented a confounding factor. The SIM− participants were exposed to the model only 5 min prior to the evaluation. Therefore, on evaluation day, they might have been surprised and experienced a lack in confidence, higher stress, and poorer performance due to ignorance of how the model would respond to their approach [17]. Nevertheless, the fact that assessment was performed 1 month after initial didactic and training reduced this *déjà-vu* phenomenon in the SIM+ group and might have counterbalanced the lack of familiarity with the task trainer of the SIM− group.

### Discussion about the primary objective

The results of the present study on the benefit of simulation-based education for surgical chest tube insertion are similar to those reporting the increase of success rate for non-surgical chest tube insertion after simulation training [15–17, 19]. One distinctive feature of the present study is to have reported that simulation increased not only the success rate but also the performance assessment score [27]. This approach could reveal that, as expected, the specific “surgical” steps of the procedure were those that benefit the most from simulation training. In fact, there was a high correlation between success and performance score. During the validation process of the performance assessment scale, a score ≥14 was always found to be associated with a functional chest tube [27].

### Discussion about secondary objectives

Simulation-based education was not associated with a decrease in procedure time for surgical chest tube insertion, as one would have expected. However, dissection time—the crucial step of the surgical procedure—significantly decreased in the SIM+ group. This implied that failure of surgical chest tube insertion was associated with poor mastery of this major step of the procedure [20–22].

Surprisingly, there was no effect of status or previous experience on the performance or success rate of surgical chest tube insertion [5, 30]. This fact might be related to the rarity of this technique (surgical approach) in France and a consequent need for adequate training, according to the recommendations [18]. Besides that, it underlines the fact that only simulation-based training was responsible for the gain in performance and success rate.

Compared with SIM− participants, SIM+ group participants judged the training model more realistic (with more anatomic features), probably because they had been trained on it repeatedly a month earlier. Similarly, there was a higher gain in self-confidence in the SIM+ group. But these differences were minimal. Indeed, self-confidence is a major issue in the performance of a stressful procedure or when the practitioner is uncomfortable with the procedure because of his/her scarce experience [11, 12]. The satisfaction rate was high in both groups—more pronouncedly in SIM+—thereby implying that participants are particularly receptive and attracted to simulation-based education as a means of improving technical skills [14, 26, 30, 31].

### External validity

We think that simulation-based education for surgical chest tube insertion can be spread to other groups of learners with the same results, in initial learning (residents) as well as continuous education (emergency physicians, intensivists) [5, 6, 13–15]. We observed that one simulation attempt did not suffice to achieve 100 % success for the whole group. This observation underscores the great interest of repeated simulation training for low-volume/high-stake procedures [20–22]. Finally, the assessment session was scheduled 1 month after the initial didactic session in both groups and simulation training in the SIM+ group. This delay might have been short as regards the occurrence of clinical surgical chest tube insertion (in our occupational field) and performance scores may have been better in simulation than in reality [16, 20, 30, 32]. Future studies should investigate if practicing on a task trainer improves the performance of the technique when performed on human subjects.

## Conclusions

Simulation-based education significantly improved the success rate and performance of surgical chest tube insertion on a traumatic pneumothorax model. This benefit was explained by a better mastery of the chest wall dissection step. It was accompanied by an increase in self-confidence contributing to performance and success. Such training appears well suited to an infrequent, difficult procedure, responsible for potential severe complications if poorly performed.

Future studies should investigate the frequency of repetition of simulation sessions as a way of sustaining the benefit of simulation-based education.
